# Comparative effectiveness of different acupuncture therapies for perimenopausal syndrome: a systematic review and network meta-analysis

**DOI:** 10.3389/fneur.2025.1696085

**Published:** 2026-01-15

**Authors:** Xiaoyan Yang, Fengya Zhu, Yue Zhong, Pengfei Liao, Qiang Ji, Siyun Li, Xia Feng, Yumiao Zheng, Qian Xue, Guiquan Chen

**Affiliations:** 1 School of Integrated Traditional Chinese and Western Medicine, The Affiliated Traditional Chinese Medicine Hospital of Southwest Medical University, Luzhou, Sichuan, China; 2 Zigong First People's Hospital, Zigong, China

**Keywords:** acupuncture, network meta-analysis, perimenopausal syndrome, randomized controlled trial, systematic review

## Abstract

**Background:**

Perimenopausal women commonly suffer from symptoms like hot flashes, insomnia, and mood swings, impacting quality of life. While acupuncture is a widely used and effective non-pharmacological treatment, the relative efficacy of different acupuncture modalities remains unclear. This study aimed to compare the effects of various acupuncture approaches on perimenopausal symptoms through a network meta-analysis.

**Methods:**

A comprehensive literature search was conducted in PubMed, Embase, Cochrane Library, Web of Science, CNKI, Wanfang, CBM, and VIP databases for randomized controlled trials (RCTs) comparing acupuncture interventions for perimenopausal syndrome, with the search updated to June 27, 2025. Risk of bias was assessed using the Cochrane tool. Network meta-analysis was performed using Stata 15 and R version 4.3.

**Results:**

A total of 49 RCTs with 4,579 participants were included. Acupuncture combined with Western medicine (AWM) was most effective for hormone regulation. Acupuncture plus Chinese medicine (ACM) best improved traditional Chinese medicine (TCM) symptoms, while electroacupuncture combined with Western medicine (EAWM) was optimal for menopausal symptoms and depression. Electroacupuncture (EA) alone was most effective for anxiety, auricular plus body acupuncture (AAA) improved sleep the most, and moxibustion (M) showed the highest overall effectiveness.

**Conclusion:**

Various acupuncture modalities show beneficial effects on perimenopausal syndrome, particularly AWM, EAWM, EA, AAA, and M. These findings provide evidence-based guidance for individualized treatment selection, although further high-quality RCTs are warranted for validation.

**Systematic Review Registration:**

https://osf.io/search, identifier 10.17605/OSF.IO/3F2GJ.

## 1 Introduction

Perimenopause refers to the transitional phase leading up to menopause and the first year following the cessation of menstruation (1). During this stage, declining ovarian function and fluctuating estrogen levels often lead to the development of perimenopausal syndrome (2). This syndrome commonly manifests through a variety of clinical symptoms, such as hot flashes, excessive sweating, urinary incontinence, joint and muscle pain, fatigue, sleep disturbances, vaginal dryness, sexual dysfunction, and sensations like formication (2, 3). As the condition progresses, it can markedly impair quality of life, contributing to increased emotional stress, social discomfort, and psychological distress (4, 5).

According to a 2012 World Health Organization (WHO) projection, the global population of perimenopausal women is expected to reach 1.2 billion by 2030, with approximately 76% residing in low- and middle-income countries (6). The perimenopausal transition is characterized by endocrine fluctuations that precipitate a spectrum of physical and psychological disturbances (7), including vasomotor symptoms (hot flushes, palpitations, night sweats, and sleep disruption), genitourinary complaints (vaginal dryness or pruritus, sexual dysfunction, and urinary symptoms), mood disorders (depression, anxiety, and irritability), and abnormal uterine bleeding (8–12). These manifestations are associated with a substantial decline in quality of life, contributing to increased familial and societal burden (13, 14).

Hormone replacement therapy (HRT) is currently regarded as the first-line treatment for perimenopausal syndrome, particularly for alleviating vasomotor symptoms, as endorsed by both international and regional clinical guidelines (15, 16). However, recent evidence indicates that HRT has been linked to an elevated risk of serious long-term adverse events, including endometrial cancer (17), breast cancer (18), stroke (19), and coronary artery disease (20). Acupuncture, a core component of traditional Chinese medicine (TCM), has been practiced in China for over 4,000 years (21) and is widely recognized for its therapeutic role in managing perimenopausal symptoms (22). It is considered one of the most accessible, safe, and widely accepted forms of complementary and alternative medicine, both in China and increasingly in Western countries (23). A recent randomized controlled trial (RCT) reported that acupuncture significantly alleviates perimenopausal symptoms and may also be effective in managing coexisting depression in affected individuals (23, 24). However, existing evidence remains insufficient to clarify the comparative effectiveness of different acupuncture modalities for the diverse manifestations of perimenopausal syndrome. Previous studies have primarily examined single techniques or limited outcomes, lacking an integrated evaluation across multiple symptom dimensions such as vasomotor instability, sleep disturbance, mood disorders, and hormonal dysregulation. To address this gap, the present study applies a network meta-analysis (NMA) to synthesize both direct and indirect evidence, enabling a systematic comparison and ranking of various acupuncture interventions. This approach aims to provide a comprehensive and evidence-based reference for optimizing individualized clinical treatment strategies.

## 2 Methods

### 2.1 Search strategy

Clinical trials published in Chinese and English up to June 17, 2025, were systematically retrieved using computerized searches. The following databases were searched: PubMed, Cochrane Library, Web of Science, EMBASE, China Biology Medicine Disc (CBM), China National Knowledge Infrastructure (CNKI), WanFang Data, and the China Science and Technology Journal Database (VIP). The search targeted studies evaluating the effects of various acupuncture interventions on multidimensional symptoms of perimenopausal syndrome.

Chinese search terms included: 针 灸 (acupuncture), 温针灸 (warm acupuncture), 电针 (electroacupuncture), 体针 (body acupuncture), 耳针 (auricular acupuncture), 艾灸 (moxibustion), 围绝经期综合征 (perimenopausal syndrome), 更年期 (menopause), and 更年 期综合征 (menopausal syndrome). English keywords included: pharmacopuncture, acupuncture treatment^*^, pharmacoacupuncture treatment, climacterium, perimenopause, and perimenopausal female.

This systematic review and network meta-analysis followed the Preferred Reporting Items for Systematic Reviews and Meta-Analyses (PRISMA) guidelines for network meta-analyses (25) and was prospectively registered in the Open Science Framework (OSF). The protocol can be accessed at https://osf.io/x6f4w (Registration doi: 10.17605/OSF.IO/3F2GJ).

### 2.2 Inclusion and exclusion criteria

#### 2.1.1 Inclusion criteria

1) Study design: RCTs investigating the use of acupuncture interventions-either alone or in combination-for the treatment of perimenopausal syndrome.
2)Participants: individuals diagnosed with perimenopausal syndrome according to recognized diagnostic criteria, including the Chinese Medical Association guidelines, the WHO diagnostic criteria for menopause, or symptom-based standards such as the Kupperman Index (KI ≥ 15). Minor variations in diagnostic approaches across studies were considered a potential source of heterogeneity.
3) Interventions: studies in which the intervention group received acupuncture-based therapies, including manual acupuncture, electroacupuncture, warm acupuncture, auricular acupuncture, or moxibustion (M), either applied alone or combined with conventional Western medicine or Chinese herbal medicine. Control groups received conventional treatment, sham acupuncture, placebo, or no treatment.
4) Outcomes:
Primary outcomes: measures assessing treatment effectiveness, including total effective rate, Kupperman Index (KI), and sleep quality scores (e.g., Pittsburgh Sleep Quality Index, PSQI).
Secondary outcomes: hormone levels [e.g., estradiol (E2), follicle-stimulating hormone (FSH), luteinizing hormone (LH)], depression/anxiety scales (e.g., HAMD, HAMA).

All outcome measures included in this review are widely recognized and validated in clinical and research settings. The KI and the PSQI have demonstrated high internal consistency and test-retest reliability (Cronbach's α > 0.80) for evaluating menopausal symptoms and sleep quality. The HAMD and HAMA scales are standardized psychological assessment tools with well-established construct validity. Hormonal assays for E2, FSH, and LH were performed using clinically validated methods, ensuring measurement reliability and reproducibility.

#### 2.1.2 Exclusion criteria

Studies not published in Chinese or English.

Trials with incomplete design or missing data that preclude estimation of effect sizes.

Studies irrelevant to the target population or interventions of interest.

Non-original research, including animal experiments, systematic reviews, meta-analyses, or case reports.

Duplicated publications or studies involving repeated data analyses.

### 2.3 Study selection and data extraction

Two reviewers independently conducted the literature search based on the predefined strategy. Initial screening was performed by evaluating titles and abstracts to exclude studies that clearly did not meet the inclusion criteria. Full texts of potentially eligible studies were then retrieved for secondary screening. Any disagreements between reviewers were resolved through discussion or adjudicated by a third researcher.

The following information was extracted from each included study: first author, publication year, country, study design, total sample size, intervention details, sex distribution, mean age, assessment tools used, and timing of outcome evaluation.

### 2.4 Quality assessment

The quality of the included studies was independently assessed by two reviewers using both the Cochrane Risk of Bias (RoB) tool and the Jadad scale. The Cochrane RoB tool evaluates studies across seven domains: random sequence generation, allocation concealment, blinding of participants and personnel, blinding of outcome assessment, completeness of outcome data, selective reporting, and other potential sources of bias. Each domain was rated as having a low, unclear, or high risk of bias.

The Jadad scale assessed methodological quality based on four criteria: randomization, allocation concealment, blinding, and withdrawals/dropouts. Studies scoring 1–3 were considered low quality, while those scoring 4–7 were regarded as high quality. Any disagreements between reviewers were resolved through discussion or consultation with a third reviewer.

### 2.5 Statistical analysis

This study commenced with a similarity assumption test to assess the clinical and methodological similarity of the included studies. The efficacy of each treatment was calculated from a frequentist perspective. For trials with three or more intervention arms, each arm was treated as an independent node within the network meta-analysis. Shared control groups were handled according to Cochrane and NMA methodological guidelines by proportionally dividing the sample size and variance to avoid double-counting participants. Pairwise effect sizes (MD or OR) were computed within each study and incorporated into the random-effects network model under the assumption of transitivity and consistency. After completing the network meta-analysis, a funnel plot was employed to evaluate visually apparent publication bias. This study utilized a random-effects model, as it is considered the most appropriate and conservative approach for analyzing between-study variance. Network graphs were constructed using STATA 15.0, providing all existing relationships and representing different treatments with various nodes. Direct comparisons of outcomes were denoted by lines connecting the appropriate nodes. The aforementioned analyses were conducted using the “coda” and “gemtc” packages in R. The rank probability of each treatment was calculated using the *p*-score, which ranges from 0 to 1, with higher values indicating better treatment efficacy.

## 3 Result

### 3.1 Literature search results

**Figure 1:**
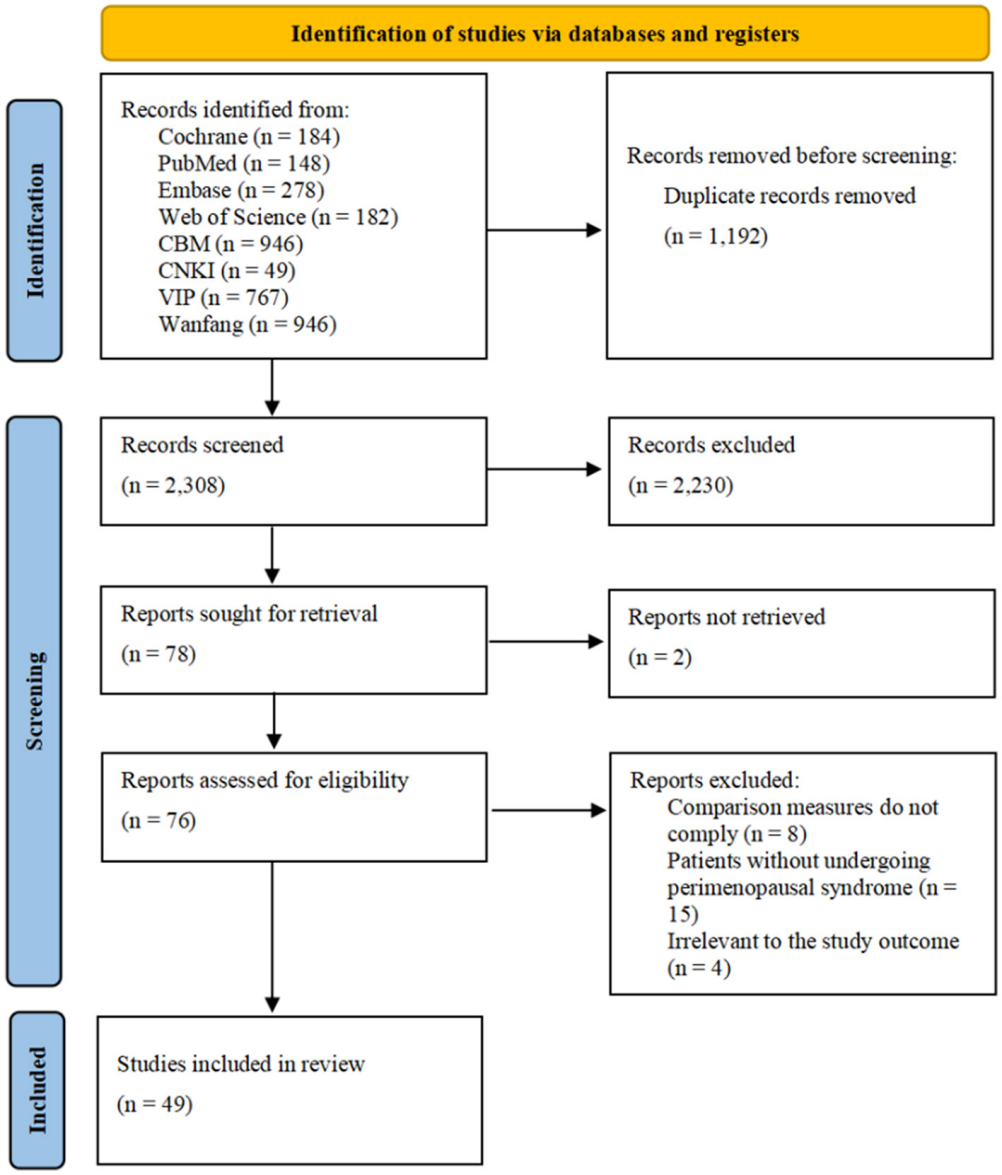
PRISMA flow diagram of study selection.

A total of 3,500 records were initially identified through comprehensive searches across multiple databases. After removing duplicates using EndNote 21, 2,308 studies remained. Following title and abstract screening, 2,230 articles were excluded. After full-text assessment, an additional 27 studies were removed based on the eligibility criteria. Ultimately, 49 studies (26–74) of moderate or high methodological quality were included in the analysis. The study selection process is illustrated in Figure 1.

### 3.2 Characteristics of included studies

A total of 49 RCTs were included in this network meta-analysis. Most studies were conducted in China (*n* = 48), with one study from Australia. The participants were predominantly perimenopausal or menopausal women diagnosed with insomnia, depression, menopausal syndrome, or traditional Chinese medicine (TCM)-defined conditions such as liver-kidney yin deficiency. The average age of participants ranged from approximately 45–55 years, and the sample size per group varied from 22 to 110, with most studies having balanced group sizes.

A wide range of interventions were examined, including manual acupuncture, electroacupuncture (EA), moxibustion, auricular point stimulation, warming acupuncture, acupoint application, and combined therapy involving acupuncture plus Western medicine. Control groups typically received Western pharmacotherapy, sham acupuncture, or usual care. Several studies included multiple intervention arms to compare the efficacy of different therapeutic modalities.

The primary outcomes evaluated across studies included total effective rate, the KI, and the PSQI. Secondary outcomes comprised hormone levels (e.g., E2, FSH, LH), psychological scales such as the Hamilton Depression Scale (HAMD) and Hamilton Anxiety Scale (HAMA), and TCM syndrome scores. Some studies assessed multiple outcomes simultaneously to provide a comprehensive evaluation of treatment efficacy.

All included studies were RCTs, and a subset explicitly reported the use of blinding methods or sham-controlled designs to enhance methodological rigor. Overall, the included literature demonstrates diversity in treatment approaches and outcome measures, providing a solid foundation for the comparative analysis of intervention effectiveness. The basic characteristics of the included studies are summarized in Supplementary Table S1.

### 3.3 Quality assessment and risk of bias evaluation

A total of 49 randomized controlled trials were included in this review. The methodological quality of each study was independently assessed using both the Jadad scale and the Cochrane Risk of Bias tool, as summarized in Supplementary Table S1 and illustrated in Supplementary Figure S1.

According to the Jadad scoring system, 45 studies scored 4 or above and were considered high quality, while the remaining four studies scored 3, indicating moderate quality. The main limitations affecting the scores were unclear descriptions of randomization procedures and insufficient implementation of blinding.

Based on the Cochrane Risk of Bias assessment, over 80% of the included trials explicitly reported appropriate methods for sequence generation. However, allocation concealment and blinding were often inadequately described or omitted, leading to a proportion of studies being rated as having unclear or high risk in these domains. Most studies were rated as low risk for incomplete outcome data and selective reporting. Overall, the included studies were of moderate to high quality, providing a relatively reliable evidence base.

### 3.4 Network meta-analysis

#### 3.4.1 Primary outcomes

##### 3.4.1.1 Total effective rate

**Figure 2:**
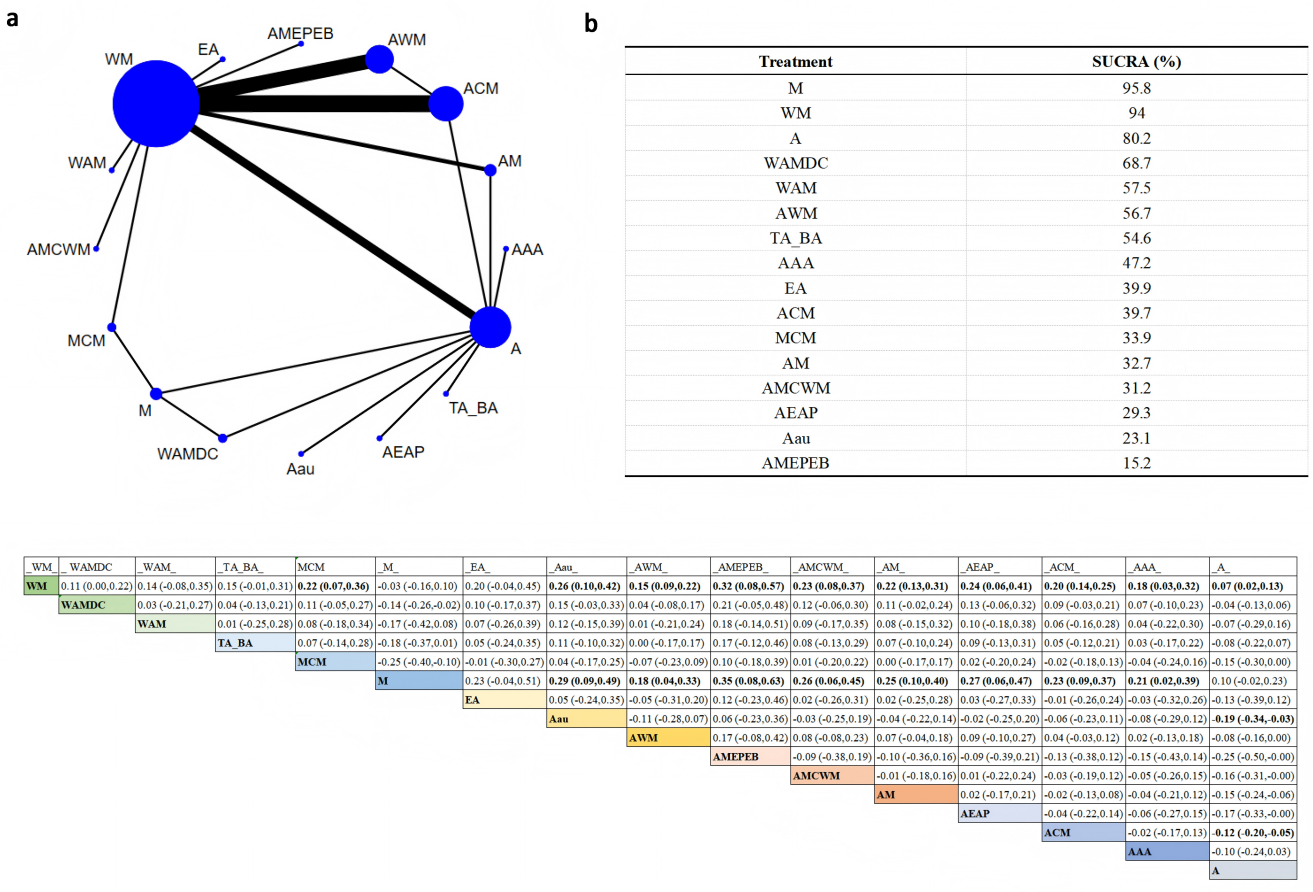
Network plot of total effective rate across interventions. **(a)** Network evidence plot **(b)** SUCRA ranking table **(c)** Pairwise comparison table.

A total of 16 different interventions were included in the network plot assessing total effective rate. In the plot, node size reflects the sample size of each intervention, and the thickness of the connecting lines indicates the number of direct comparisons between interventions (Figure 2).

The SUCRA rankings indicated that intervention M had the highest probability of being the most effective (SUCRA = 95.8%), followed by WM (SUCRA = 94.0%) and A (SUCRA = 80.2%). According to the league table, intervention M showed no statistically significant differences compared to WM and A. However, M was significantly superior to Aau (RR = 0.29, 95% CI: 0.09–0.49), Acupuncture combined with Western medicine (AWM; RR = 0.18, 95% CI: 0.04–0.33), AMEPEB (RR = 0.35, 95%CI: 0.08–0.63), AMCWM (RR = 0.26, 95% CI: 0.06–0.45), AM (RR = 0.25, 95%CI: 0.10–0.40), AEAP (RR = 0.27, 95% CI: 0.06–0.47), Acupuncture plus Chinese medicine (ACM; RR = 0.23, 95% CI: 0.09–0.37), and auricular plus body acupuncture (AAA; RR = 0.21, 95% CI: 0.02–0.39).

These findings suggest that intervention M may offer superior efficacy in improving the total effective rate and may be considered as a preferred option in clinical settings.

##### 3.4.1.2 Kupperman index

**Figure 3:**
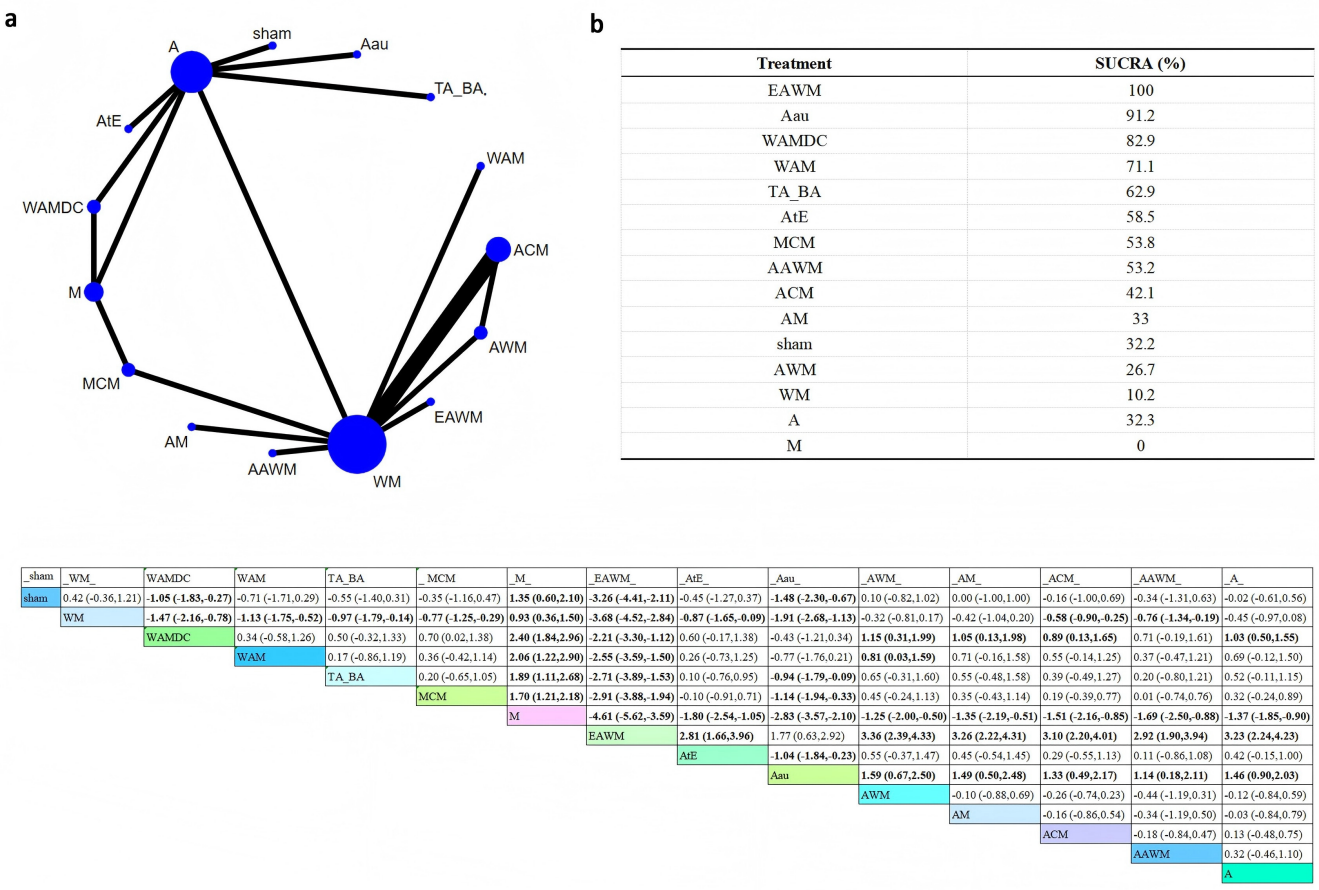
Network plot of Kupperman Index scores. **(a)** Network evidence plot **(b)** SUCRA ranking table **(c)** Pairwise comparison table.

The network plot for KI included 15 interventions, and the overall network structure was relatively well-connected and balanced (Figure 3).

SUCRA rankings showed that electroacupuncture combined with Western medicine (EAWM) had the highest probability of being the most effective (SUCRA = 100%), followed by Aau (SUCRA = 91.2%).

The league table demonstrated that EAWM was significantly more effective than sham (SMD = −3.26, 95%CI: −4.41 to −2.11), WM (SMD = −3.68, 95%CI: −4.52 to −2.84), WAMDC (SMD = −2.21, 95%CI: −3.30 to −1.12), WAM (SMD = −2.55, 95%CI: −3.59 to −1.50), TA_BA (SMD = −2.71, 95%CI: −3.89 to −1.53), MCM (SMD = −2.91, 95%CI: −3.88 to −1.94), and M (SMD = −4.61, 95%CI: −5.62 to −3.59).

These results suggest that EAWM may be the most effective intervention for alleviating menopausal symptoms measured by the Kupperman Index.

##### 3.4.1.3 Pittsburgh sleep quality index

**Figure 4:**
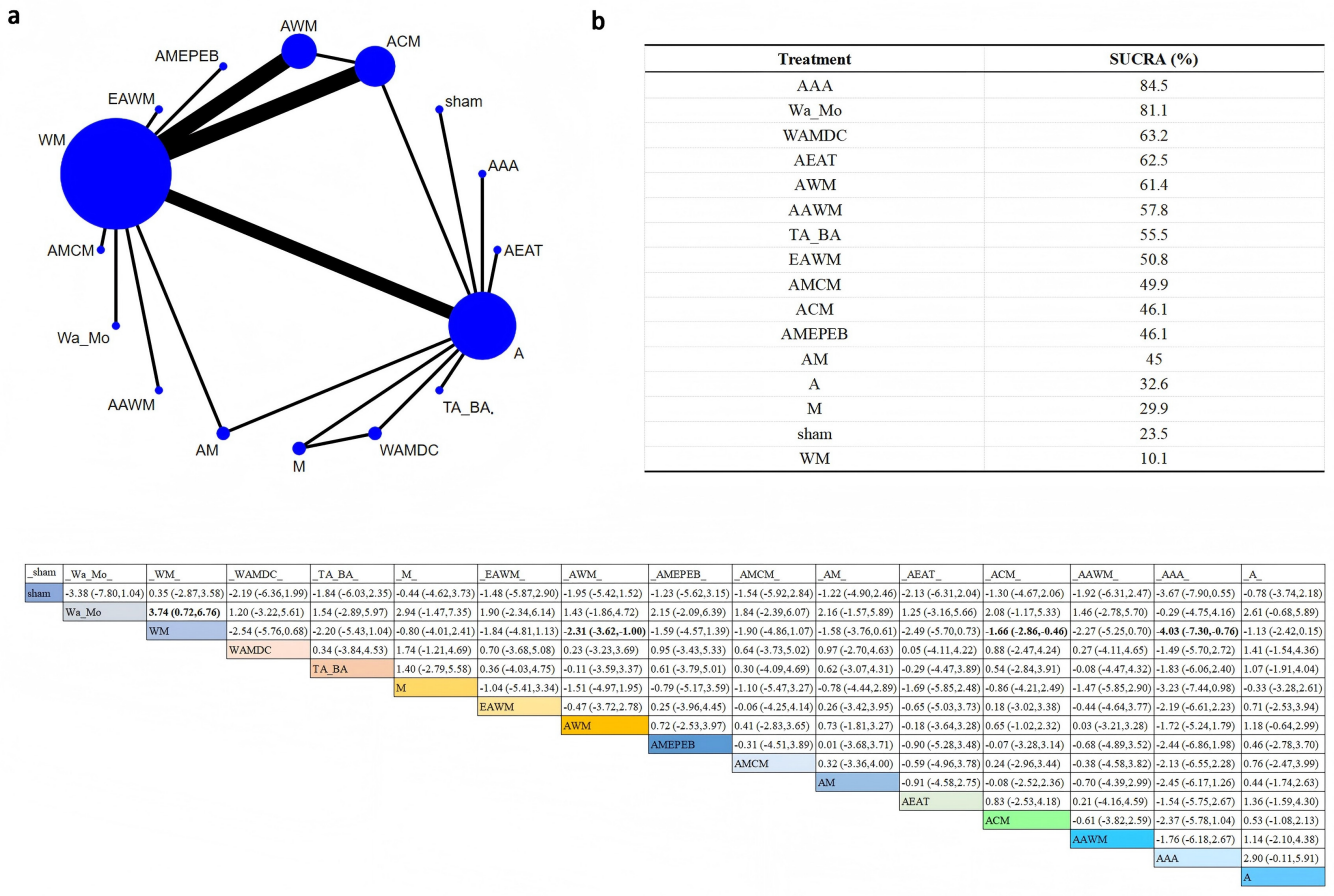
Network plot of PSQI. **(a)** Network evidence plot **(b)** SUCRA ranking table **(c)** Pairwise comparison table.

In the network plot for PSQI, several interventions-WM, AWM, ACM, A, WAMDC, M, and AM-were directly or indirectly compared (Figure 4).

According to SUCRA rankings, AAA was most likely to be the best intervention (SUCRA = 84.5%) in improving sleep quality, followed closely by Wa_Mo (SUCRA = 81.1%).

The league table showed that AAA was significantly more effective than WM (SMD = −4.03, 95% CI: −7.30 to −0.76). In addition, AWM (SMD = −2.31, 95% CI: −3.62 to −1.00) and ACM (SMD = −1.66, 95% CI: −2.86 to −0.46) were also superior to WM.

These findings suggest that AAA may be the most effective intervention for improving sleep quality in this population.

#### 3.4.2 Secondary outcomes

##### 3.4.2.1 Hormone levels

**Figure 5:**
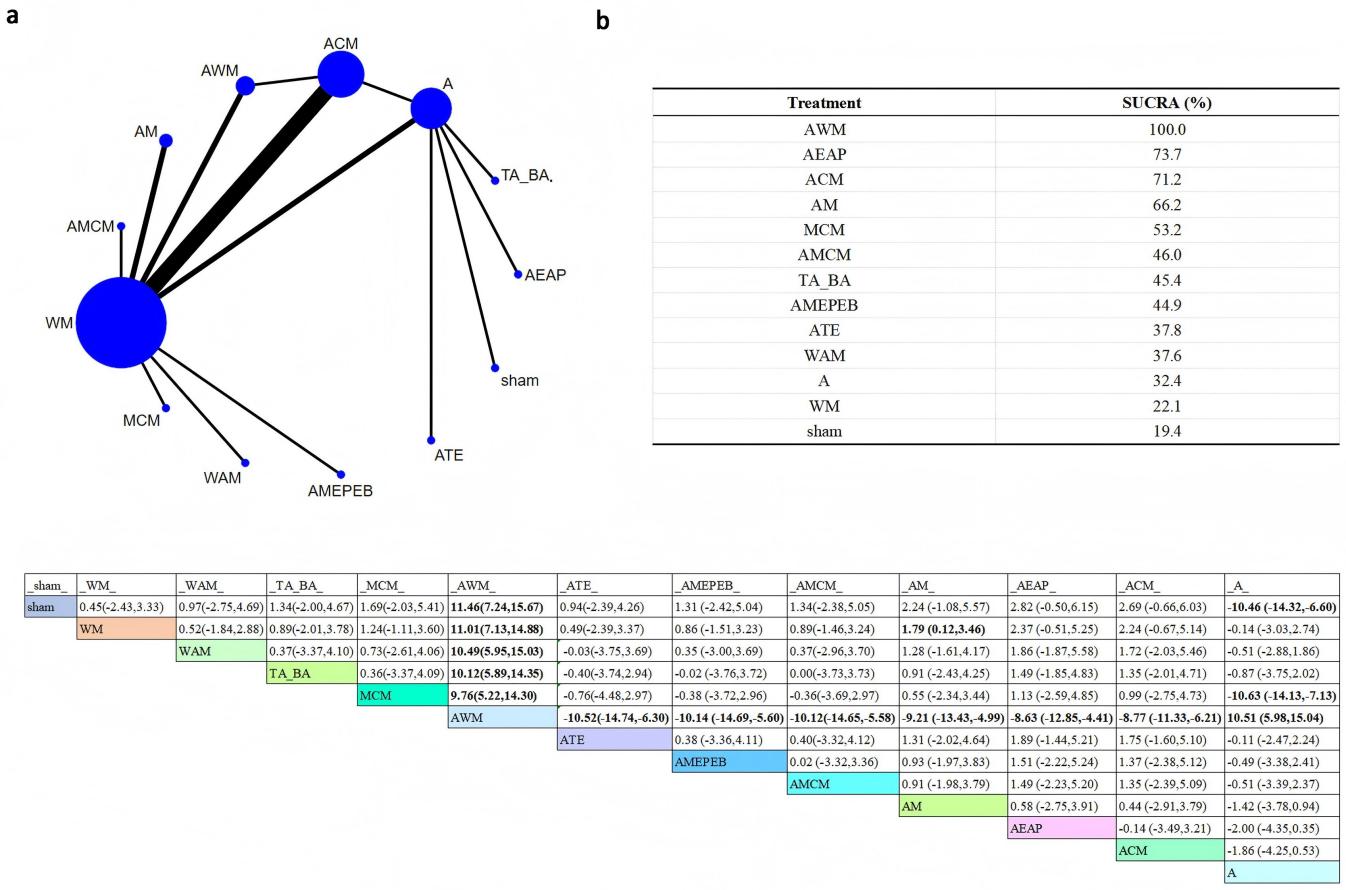
Network plot for E2 level comparisons. **(a)** Network evidence plot **(b)** SUCRA ranking table **(c)** Pairwise comparison table.

**Figure 6:**
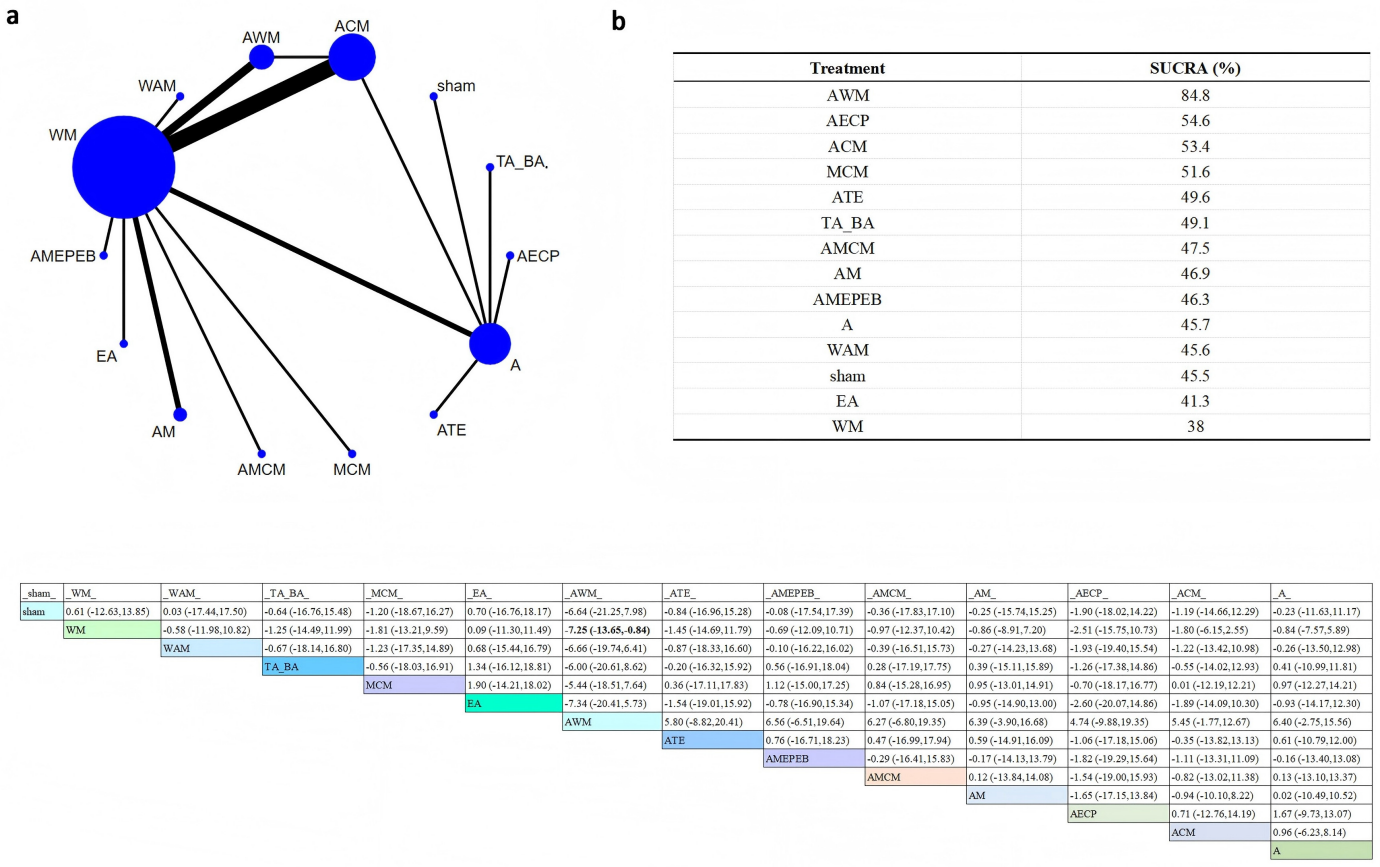
Network plot for FSH levels. **(a)** Network evidence plot **(b)** SUCRA ranking table **(c)** Pairwise comparison table.

**Figure 7:**
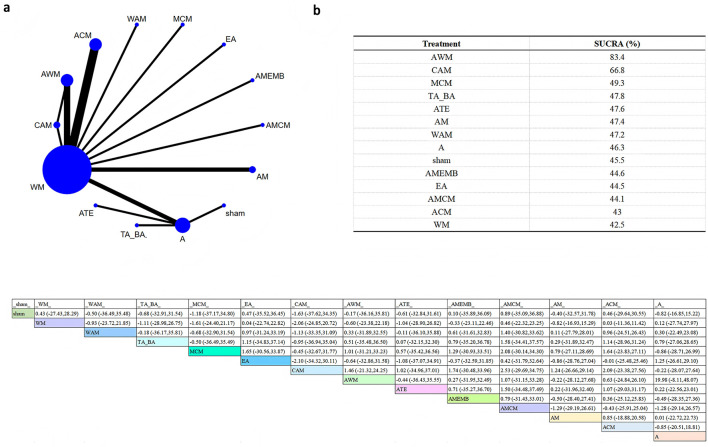
Network plot for LH levels. **(a)** Network evidence plot **(b)** SUCRA ranking table **(c)** Pairwise comparison table.

Three hormonal indicators were analyzed: estradiol (E2; Figure 5), follicle-stimulating hormone (FSH; Figure 6), and luteinizing hormone (LH; Figure 7). The network plot showed that interventions including AWM, ACM, AM, MCM, AMCM, TA_BA, ATE, WAM, A, WM, and sham were evaluated in hormone-related studies (Figures 5–7).

SUCRA analysis revealed that AWM was most effective in increasing E2 levels (SUCRA = 100%), and also showed high efficacy in reducing FSH (SUCRA = 84.8%) and LH (SUCRA = 83.4%).

According to the league table, AWM significantly increased E2 levels compared to sham (SMD = 11.46, 95%CI: 7.24–15.67), WM (SMD = 11.01, 95%CI: 7.13–14.88), WAM (SMD = 10.49, 95%CI: 5.95–15.03), TA_BA (SMD = 10.12, 95%CI: 5.89–14.35), and MCM (SMD = 9.76, 95%CI: 5.22–14.30). Compared with WM, AWM also significantly reduced FSH (SMD = −7.25, 95%CI: −13.65 to −0.84). No statistically significant differences were observed between interventions in terms of LH. These results support the potential endocrine-regulating effect of AWM.

##### 3.4.2.2 Depression and anxiety scales (HAMD/HAMA)

**Figure 8:**
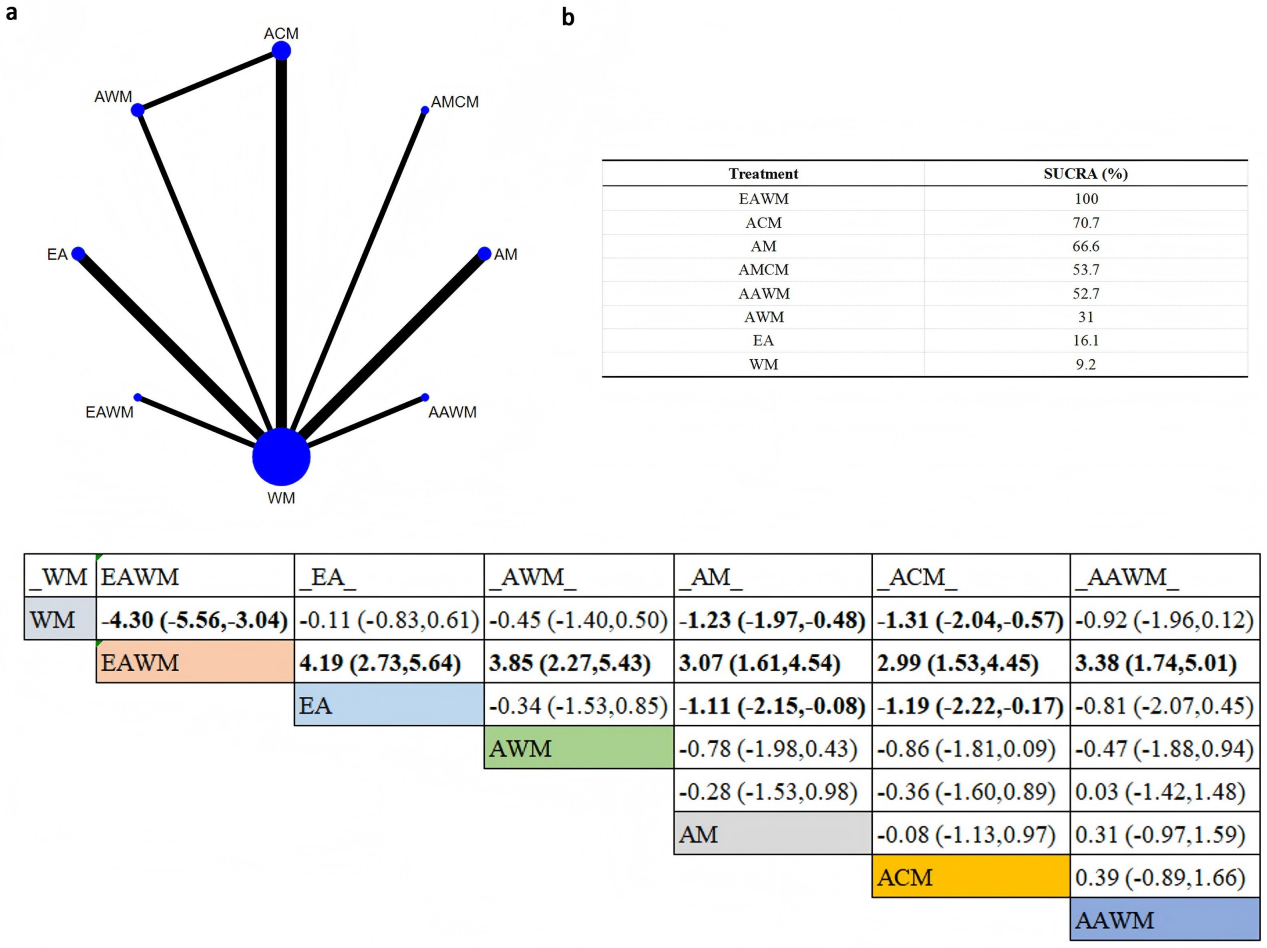
Network plot for HAMD scores. **(a)** Network evidence plot **(b)** SUCRA ranking table **(c)** Pairwise comparison table.

The network plot showed that eight interventions were evaluated for HAMD (Figure 8) and seven for HAMA (Supplementary Figure S2). A closed loop was formed among WM, AWM, and ACM, indicating the presence of indirect comparisons.

According to SUCRA rankings, EAWM was the most effective intervention for reducing HAMD scores (SUCRA = 100%), followed by ACM (SUCRA = 70.7%). For HAMA, the top-ranking intervention was EA (SUCRA = 99.6%), followed by ACM (SUCRA = 68.3%).

The league table showed that EAWM was significantly superior to ACM in reducing HAMD (MD = 2.99, 95%CI: 1.53–4.45). Furthermore, ACM (MD = −1.19, 95%CI: −2.22 to −0.17) and AM (MD = −1.11, 95%CI: −2.15 to −0.08) were significantly better than EA in improving depressive symptoms. No significant differences were observed between interventions for HAMA scores. These findings suggest that EAWM may be particularly effective in alleviating perimenopausal depressive and anxiety symptoms.

##### 3.4.2.3 TCM syndrome score

A total of seven interventions were assessed for their effects on TCM syndrome scores (Supplementary Figure S3). According to SUCRA rankings, ACM had the highest probability of being the most effective (SUCRA = 55.2%), followed closely by AEAP (SUCRA = 55.0%).

However, the league table showed no statistically significant differences between the seven interventions in improving TCM syndrome scores.

#### 3.4.3 Publication bias

Comparison-adjusted funnel plots revealed potential publication bias across all four assessed outcomes (Supplementary Figure S4). The overall risk of bias in the included randomized controlled trials was judged as presenting “some concerns,” indicating a possible influence of publication bias or small-study effects.

## 4 Discussion

### 4.1 Summary of main findings

This study was designed to address a key clinical issue in the management of perimenopausal syndrome, how to determine the most effective and evidence-based acupuncture modality among the diverse therapeutic options currently applied in practice. Although acupuncture is widely used to relieve perimenopausal symptoms, the absence of standardized treatment selection and the inconsistency of therapeutic outcomes across different modalities have limited its clinical guidance value. Existing studies have largely focused on single interventions or isolated outcomes, without providing a comprehensive comparison across the multidimensional manifestations of perimenopausal syndrome, such as hormonal imbalance, sleep disturbance, and mood disorders. In this context, the present network meta-analysis systematically compared the efficacy of multiple acupuncture-related interventions to address these gaps. The findings indicated that intervention M may be more useful in improving the total effective rate, while EAWM ranked highest, suggesting that it may be more useful in alleviating menopausal symptoms measured by the Kupperman Index. For sleep quality, AAA appeared to provide greater improvement in PSQI scores, and AWM tended to yield more notable effects on hormonal regulation, particularly in increasing E2 and reducing FSH. Regarding mood disorders, EAWM appeared to be more useful in reducing HAMD scores, whereas EA tended to perform better in alleviating anxiety as reflected by HAMA. Although ACM achieved the highest ranking for TCM syndrome scores, no statistically significant differences were observed among the various interventions.

### 4.2 Comparison with existing literature

The onset of perimenopausal syndrome is closely associated with a combination of physiological and psychological factors (75, 76). As ovarian function gradually declines, fluctuations in estrogen and progesterone levels become more pronounced, disrupting reproductive and endocrine homeostasis. These hormonal changes can lead to a range of neuropsychiatric symptoms such as insomnia, anxiety, and depression (77, 78), and may significantly impair daily functioning and quality of life in more severe cases.

Sleep regulation involves coordinated control by both lower centers (e.g., hypothalamus) and higher cortical centers. Hormonal fluctuations can impair cortical modulation of sleep-related brain regions, leading to disrupted circadian rhythms and altered sleep architecture, which in turn may trigger or exacerbate insomnia (79, 80).

Moreover, the decline in estrogen levels may inhibit the synthesis and release of key neurotransmitters in the central nervous system, particularly serotonin (5-HT) and gamma-aminobutyric acid (GABA), both of which are essential for emotional stability and sleep regulation (80). A deficiency in 5-HT is associated with depressed mood and difficulty falling asleep, while reduced GABA levels increase central nervous excitability, further compromising sleep quality. Therefore, fluctuations in hormone levels contribute to perimenopausal sleep and mood disorders through neuroendocrine and neurotransmitter-mediated pathways.

Our findings are consistent with previous meta-analyses suggesting that multimodal interventions, especially those integrating acupuncture and herbal medicine, demonstrate significant improvements in perimenopausal symptoms. For instance, a meta-analysis by Jiang et al. (81) highlighted that acupuncture combined with Western medicine was superior to monotherapies in alleviating menopausal symptoms, aligning with our findings for KI and mood symptoms. Additionally, current evidence suggests that integrative therapy involving acupuncture and Chinese herbal medicine may offer superior benefits over Western medicine alone in enhancing sleep quality and modulating FSH levels in individuals with perimenopausal insomnia (82). The research indicated that electroacupuncture appeared to be more useful in reducing KI scores than conventional medication or sham electroacupuncture, indicating their potential advantages in alleviating menopausal symptoms (83). Moreover, acupuncture appears to be more useful than conventional hypnotics in the management of perimenopausal insomnia and may concurrently relieve associated menopausal symptoms such as mood disturbances and vasomotor instability (21, 84). The underlying mechanisms of acupuncture are multifaceted, encompassing anti-inflammatory and antioxidative effects, enhancement of endogenous pain inhibitory pathways, promotion of lipolysis, immunomodulation, and regulation of neurotransmitters in the central nervous system (85, 86).

In terms of depression and anxiety, the superior performance of EAWM and EA may be explained by mechanisms such as regulation of the hypothalamic-pituitary-adrenal (HPA) axis, enhancement of serotonin activity, and modulation of inflammatory cytokines, which have been well-documented in animal and clinical studies (87).

The lack of significant differences among TCM syndrome interventions may reflect both the subjective nature of TCM syndrome scoring and potential heterogeneity in syndrome classification across trials.

Compared with previous studies that focused on single acupuncture techniques or a limited range of clinical outcomes, the present study incorporated multiple acupuncture-based interventions and evaluated a broader spectrum of perimenopausal symptoms within one analytical framework. By using a network meta-analysis approach, both direct and indirect evidence were synthesized to generate a comparative ranking of treatment efficacy, allowing for a more integrated understanding of therapeutic differences among modalities. In contrast to earlier research that primarily addressed symptom alleviation, this study simultaneously examined hormonal regulation, emotional wellbeing, and sleep quality, thereby offering a more comprehensive assessment of clinical benefit. The innovation of this work lies in combining the conceptual principles of Traditional Chinese Medicine with modern evidence-based methodology to establish an efficacy hierarchy among diverse acupuncture strategies. Moreover, the finding that electroacupuncture combined with Western medicine may achieve superior outcomes provides a new perspective for developing individualized, multimodal interventions tailored to the complex needs of perimenopausal women.

### 4.3 Strengths and limitations

This study is the first to comprehensively compare multiple traditional and integrative therapies for perimenopausal symptoms across several outcome domains using network meta-analysis. By employing SUCRA rankings and league tables, we provide a hierarchy of interventions that is clinically informative.

However, some limitations must be acknowledged. First, although most trials were rated as having some concerns regarding bias, the possibility of publication bias and small-study effects cannot be excluded. Second, heterogeneity in intervention protocols and outcome definitions may have introduced inconsistencies. Third, many studies had relatively short follow-up periods, limiting our ability to assess long-term effects. Fourth, the literature search was restricted to studies published in English and Spanish, and gray literature such as conference abstracts, dissertations, and non-indexed reports were not included. These restrictions may have resulted in the omission of relevant studies published in other languages or sources, introducing potential language and publication bias. As studies with negative or non-significant results are more likely to remain unpublished, the exclusion of gray literature may have led to an overestimation of treatment effects.

### 4.4 Implications for practice and research

Our findings suggest that integrated interventions, particularly EAWM and AWM, may be considered in clinical decision-making for perimenopausal symptom management. Future studies should aim for higher methodological rigor, standardized outcome measures, and longer follow-up durations. Moreover, mechanistic studies are warranted to further elucidate the biological basis underlying the observed clinical benefits.

This review has practical value for clinicians, researchers, and patients. By ranking acupuncture modalities, it offers clinicians an evidence-based guide to selecting individualized interventions for perimenopausal symptoms, improving treatment precision and outcomes in routine care. It also delineates key evidence gaps, including limited long-term data, heterogeneous outcome measures, and underrepresentation of non-Asian populations, thereby orienting researchers toward multicenter trials, standardized endpoints, and mechanistic studies. For patients, the synthesis clarifies the relative benefits of available acupuncture options, supporting informed choices about complementary and integrative treatments consistent with personal preferences and safety.

In summary, this network meta-analysis suggests potential benefits of multimodal and integrative therapies in managing various dimensions of perimenopausal symptoms and offers a basis for evidence-based clinical application.

## Data Availability

The original contributions presented in the study are included in the article/Supplementary material, further inquiries can be directed to the corresponding author.
