# Supplementary material for: Comparative effectiveness of different acupuncture therapies for perimenopausal syndrome: a systematic review and network meta-analysis
Source: Front Neurol. 2026 Jan 15;16:1696085. doi: 10.3389/fneur.2025.1696085 (PMC12852026; doi:10.3389/fneur.2025.1696085)
Supplement: Supplementary file 1 [file Table_1.docx]

**Supplementary Table 1** Essential features of the included studies

| **study** | **type** | **country** | **patient** | **treatment** | | | **outcome** | **Jadad** |
| --- | --- | --- | --- | --- | --- | --- | --- | --- |
|  |  |  |  | **name** | **n** | **age** |  |  |
| Xin Liu 2023 | RCT | China | perimenopausal women | MA | 56 | 49.00±3.704 | HAMA、GAD-7、PQSI | 7 |
|  |  |  |  | Acupuncture | 56 | 50±2.963 |  |  |
| Fei-Yi Zhao 2023 | RCT | Australia. | perimenopause | Acupuncture | 35 | 48.94 ± 2.25 | HAM-D 17、PSQI、HAM-A、KI、ISI、MenQoL | 6 |
|  |  |  |  | sham | 35 | 48.80 ± 2.07 |  |  |
| Jun-He Zhou 2022 | RCT | China | Perimenopausal Depression | EA | 108 | 45~55 | MenQoL、HAMD-17、FSH、E2、LH | 6 |
|  |  |  |  | western medicine | 104 |  |  |  |
| Shanshan L 2022 | RCT | China | Perimenopausal Insomnia | Acupuncture | 42 | 52.12±4.19 | PSQI、Men-QoL、ISI, SAS and SDS | 6 |
|  |  |  |  | sham | 42 | 53.07±3.81 |  |  |
| YANG Wenjia 2024 | RCT | China | perimenopausal insomnia | Acupuncture | 43 | 51.4±6.7 | PSQI、ISI、BAD、BAI、FSH、E2、LH | 5 |
|  |  |  |  | sham | 45 | 52.2±6.2 |  |  |
| Jing An 2021 | RCT | China | perimenopausal insomnia | Acupuncture_Chinese_medicine | 40 | 50.10±2.20 | PSQI、effect | 4 |
|  |  |  |  | Western medicine | 40 | 50.12±2.23 |  |  |
| Jiaoxiang Wang 2024 | RCT | China | perimenopausal insomnia | Acupuncture | 31 | 51.34±4.15 | PSQI、effect、TCM Syndrome Score积分、FSH、E2 | 5 |
|  |  |  |  | Acupuncture_Chinese_medicince | 31 | 51.05±4.66 |  |  |
| Liuyan Jing 2020 | RCT | China | perimenopausal insomnia | Acupuncture_Western_medicince | 43 | 51.14±2.98 | PSQI、effect | 5 |
|  |  |  |  | Western medicine | 43 | 52.46±3.24 |  |  |
| Qingpu Dong 2020 | RCT | China | perimenopausal insomnia | Acupuncture_Western_medicince | 45 | 51.21±2.12 | effect、PQSI、LH、FSH | 4 |
|  |  |  |  | Western medicine | 45 | 51.46±2.27 |  |  |
| Qingyun Guan 2020 | RCT | China | perimenopausal insomnia | Acupuncture_Western_medicince | 60 | ５０.３ ± ２.６ | effect | 4 |
|  |  |  |  | Western medicine | 60 | ５１ ．６ ± ２ ．４ |  |  |
| Mengying Li 2023 | RCT | China | perimenopausal insomnia | Acupuncture_ear_acupoint_pressure | 60 | 50.31±4.26 | PSQI、effect、TCM Syndrome Score、FSH、E2、ISI | 4 |
|  |  |  |  | Acupuncture | 59 | 51.42±4.23 |  |  |
| Wenqin Lin 2022 | RCT | China | perimenopausal insomnia | Acupuncture_Chinese_medicine | 52 | 49.64±1.13 | PSQI、KI、MenQoL、 | 4 |
|  |  |  |  | Western medicine | 52 | 49.31±1.09 |  |  |
| Huixiu Qiao 2022 | RCT | China | perimenopausal insomnia | Acupuncture_Chinese_medicine | 35 | 48.6±1.5 | effect、PSQI、SF-36 | 4 |
|  |  |  |  | Western medicine | 35 | 48.2±1.0 |  |  |
| Na Lu 2023 | RCT | China | perimenopausal insomnia | Acupuncture | 49 | 48.12±2.49 | effect、PSQI、KI | 4 |
|  |  |  |  | Acupuncture_auriculotherapy | 49 | 48.26±2.54 |  |  |
| Hongxia Liu 2024 | RCT | China | menopausal syndrome | Acupuncture_Chinese_medicine | 30 | 48±2.638 | effect、PSQI、KI、LH、FSH、E2 | 5 |
|  |  |  |  | Western medicine | 30 | 48.14±2.417 |  |  |
| Qin Guo 2021 | RCT | China | perimenopausal insomnia | Acupuncture | 30 | 9.83±3.65 | effect、PSQI、KI、E2 | 4 |
|  |  |  |  | Western medicine | 30 | 50.20±4.10 |  |  |
| Ancui Huang 2024 | RCT | China | climacteric syndrome | moxibustion _Chinese_medicine | 43 | 52.97±4.88 | effect、TCM Syndrome Score、KI、LH、FSH、E2 | 4 |
|  |  |  |  | Western medicine | 43 | 53.44±5.19 |  |  |
| Ping Li 2020 | RCT | China | Perimenopausal Depression | EA | 30 | 49±3 | effect、HAMD、HAMA | 4 |
|  |  |  |  | Western medicine | 30 | 51±3 |  |  |
| Xi Zhang 2020 | RCT | China | Perimenopausal Depression | Acupuncture_moxibustion | 30 | 50±3 | HAMD、KI、effect | 5 |
|  |  |  |  | Western medicine | 30 | 49±2 |  |  |
| Min Liu 2024 | RCT | China | perimenopausal insomnia | Acupuncture_moxibustion | 51 | 47.94±3.75 | effect、PSQI、LH、FSH、E2、 | 4 |
|  |  |  |  | Western medicine | 51 | 48.77±3.46 |  |  |
| Biru Peng 2021 | RCT | China | perimenopausal insomnia | Acupuncture_Chinese_medicine | 38 | 50.03±3.47 | PSQI、HAMD、HAMA | 3 |
|  |  |  |  | Western medicine | 38 | 50.08±3.52 |  |  |
| Qian Tang 2024 | RCT | China | Climacteric Syndrome | Acupuncture_Moxibustion_Chinese_Western_medicine | 55 | 49.56±2.45 | effect、PSQI、HAMA、HAMD | 4 |
|  |  |  |  | Western medicine | 55 | 48.47±2.02 |  |  |
| Gusheng Ran 2022 | RCT | China | perimenopausal insomnia | Acupuncture_Moxibustion_Chinese_medicine | 43 | 50.63±7.59 | PSQI、ISI、LH、FSH、E2 | 4 |
|  |  |  |  | Western medicine | 43 | 50.51±7.57 |  |  |
| Yongzhong Wang 2021 | RCT | China | Perimenopausal Syndrome | Acupuncture_Chinese_medicine | 110 | 51.39±3.41 | effect、TCM Syndrome Score、SAS、SDS、FSH、LH、E2 | 4 |
|  |  |  |  | Western medicine | 110 | 51.24±3.27 |  |  |
| Xinchang Wei 2024 | RCT | China | perimenopausal insomnia | TA_BA． | 29 | 50.071±2.963 | effect、PSQI、KI、FSH、LH、E2 | 5 |
|  |  |  |  | Acupuncture | 28 | 51．20±2．62 |  |  |
| Juxing Wu 2022 | RCT | China | menopausal insomnia | Acupuncture_Western_medicine | 50 | 47.7±2.9 | effect、FSH、LH、E2 | 4 |
|  |  |  |  | Western medicine | 50 | 47.0±3.0 |  |  |
| Huaping Xiang | RCT | China | menopausal insomnia | Acupuncture | 56 | 48.8±5.8 | effect、PSQI、FSH、LH、E2 | 4 |
|  |  |  |  | Western medicine | 56 | 49.2±6.1 |  |  |
| Kuijun Xu 2023 | RCT | China | perimenopausal insomnia | Acupuncture_Chinese_medicine | 31 | 51.82±3.94 | effect、PSQI、FSH、LH、E2 | 5 |
|  |  |  |  | Western medicine | 31 | 50.88±4.88 |  |  |
| Wenyan Xuanyuan 2020 | RCT | China | perimenopausal insomnia | Acupuncture_Western_medicine | 22 | 51.52±4.37 | effect、PSQI | 4 |
|  |  |  |  | Western medicine | 22 | 51.42±4.21 |  |  |
| Wenjia Yang 2023 | RCT | China | perimenopausal insomnia | Acupuncture | 35 | 53±4 | PSQI | 6 |
|  |  |  |  | sham | 35 | 52±4 |  |  |
| Mei Yu 2021 | RCT | China | perimenopausal insomnia | Acupuncture_Chinese_medicine | 75 | 58.34±7.13 | effect、FSH、E2 | 4 |
|  |  |  |  | Western medicine | 75 | 59.38±6.21 |  |  |
| Meng Zhao 2023 | RCT | China | perimenopausal insomnia | Acupuncture | 40 | 49.85±2.46 | effect、PSQI、FSH、LH、E2、TCM Syndrome Score | 4 |
|  |  |  |  | Western medicine | 40 | 50.1 7±2.71 |  |  |
| Jie zhou 2024 | RCT | China | perimenopausal insomnia | moxibustion | 36 | 57.41±1.45 | PSQI | 4 |
|  |  |  |  | Western medicine | 36 | 57.52±1.49 |  |  |
| Jiafang Zhu 2022 | RCT | China | perimenopausal insomnia | Acupuncture | 27 | 50±3 | PSQI、effect | 5 |
|  |  |  |  | Western medicine | 26 | 50±2.963 |  |  |
| Jingbin Chen 2024 | RCT | China | perimenopausal insomnia,Anxiety and Depression | Acupuncture_moxibustion_ear point embedding beans | 30 | 48.69±1.58 | effect、PSQI、SAS、SDS、FSH、E2 | 4 |
|  |  |  |  | Western medicine | 30 | 48.75±1.62 |  |  |
| Pei Luo 2022 | RCT | China | perimenopausal syndrome | WA | 56 | 49.29±3.99 | MRS、KI | 5 |
|  |  |  |  | AA | 59 | 50.17±4.52 |  |  |
|  |  |  |  | WA_AA | 57 | 50.82±4.5 |  |  |
| Si Chen 2024 | RCT | China | perimenopausal insomnia,Anxiety and Depression | Acupuncture_moxibustion | 60 | 50.64±1.65 | effect、PSQI、HADS、SF-36 | 5 |
|  |  |  |  | Acupuncture | 40 | 50.94±1.78 |  |  |
| Wei Dai 2022 | RCT | China | perimenopausal depression | EA_Western_medicine | 38 | 48.68±2.28 | HAMD-17、KI、PSQI、HAMS | 4 |
|  |  |  |  | Western_medicine | 35 | 48.51±3.32 |  |  |
| Xuan Zhai 2024 | RCT | China | menopausal syndrome | moxibustion_Chinese_medicine | 60 | 50.10±4.70 | effect、FSH、LH、KI、MRS | 4 |
|  |  |  |  | moxibustion | 60 | 51.10±4.60 |  |  |
| Heqing Lu 2020 | RCT | China | perimenopausal insomnia,Anxiety and Depression | Acupuncture_AA | 30 | 48.3+5.0 | effect、PSQI、KI、TCM Syndrome Score、 | 3 |
|  |  |  |  | Acupuncture | 30 | "48.6+4.8 |  |  |
| Xiaoying Hu 2024 | RCT | China | perimenopausal syndrome | acupoint thread embedding | 38 | 51±6 | KI、CMRS 35、FSH、E2、LH | 6 |
|  |  |  |  | Acupuncture | 37 | 51±5 |  |  |
| Hualei Zhou 2024 | RCT | China | perimenopausal insomnia | WA_moxibustion_drug cupping | 52 |  | effect、PSQI、KI | 4 |
|  |  |  |  | Acupuncture | 52 |  |  |  |
|  |  |  |  | moxibustion | 52 |  |  |  |
| Huan Feng 2022 | RCT | China | perimenopausal insomnia | auricular point seed burying | 35 | 51.03 ± 3.94 | PSQI、SAS、SDS、effects | 3 |
|  |  |  |  | auricular point seed burying_moxibustion | 35 | 50.91 ± 4.45 |  |  |
| Wanze Li 2020 | RCT | China | perimenopausal syndrome | WA_moxibustion | 35 | 45.78±12.83 | effect、KI、MenQoL、FSH、LH、E2 | 4 |
|  |  |  |  | Western_medicine | 35 | 46.47±14.20 |  |  |
| Juan Liu 2023 | RCT | China | perimenopausal insomnia | AA_Western_medicine | 39 | 49.5±3.0 | PSQI、HAMA、HAMD、TCM Syndrome Score | 4 |
|  |  |  |  | Western_medicine | 40 | 49.6±2.7 |  |  |
| Yuchen Xie 2022 | RCT | China | perimenopausal insomnia | Acupuncture_moxibustion | 47 | 6.44±3.50 | ESS、PSQI、HAMA、FSH、LH、E2 | 4 |
|  |  |  |  | Western_medicine | 46 | 46.37±3.48 |  |  |
| Mian Li 2022 | RCT | China | menopausal depression | Acupuncture_Western_medince | 50 | 49.20 ± 2.02 | HAMA、effect、HAMD、KI、PSQI、FSH、LH、E2 | 4 |
|  |  |  |  | Western_medicine | 50 | 49.68 ± 2.10 |  |  |
| Xiaojing Zhang 2024 | RCT | China | perimenopausal insomnia | Acupuncture_Western_medince | 35 | 45．69±4．7 | effect、PSQI、HAMA | 3 |
|  |  |  |  | Western medicine | 35 | 44．78土5．2 |  |  |
| Xueli Yan 2020 | RCT | China | perimenopausal insomnia | Acupuncture_Chinese_medicine | 59 | 50.8±7.6 | effect、ESS、PSQI、HAMA、TCM Syndrome Score、FSH、LH、E2 | 4 |
|  |  |  |  | Western medicine | 57 | 49.6±7.2 |  |  |
