# Supplementary figures and images for: Comparative effectiveness of different acupuncture therapies for perimenopausal syndrome: a systematic review and network meta-analysis
Source: Front Neurol. 2026 Jan 15;16:1696085. doi: 10.3389/fneur.2025.1696085 (PMC12852026; doi:10.3389/fneur.2025.1696085)

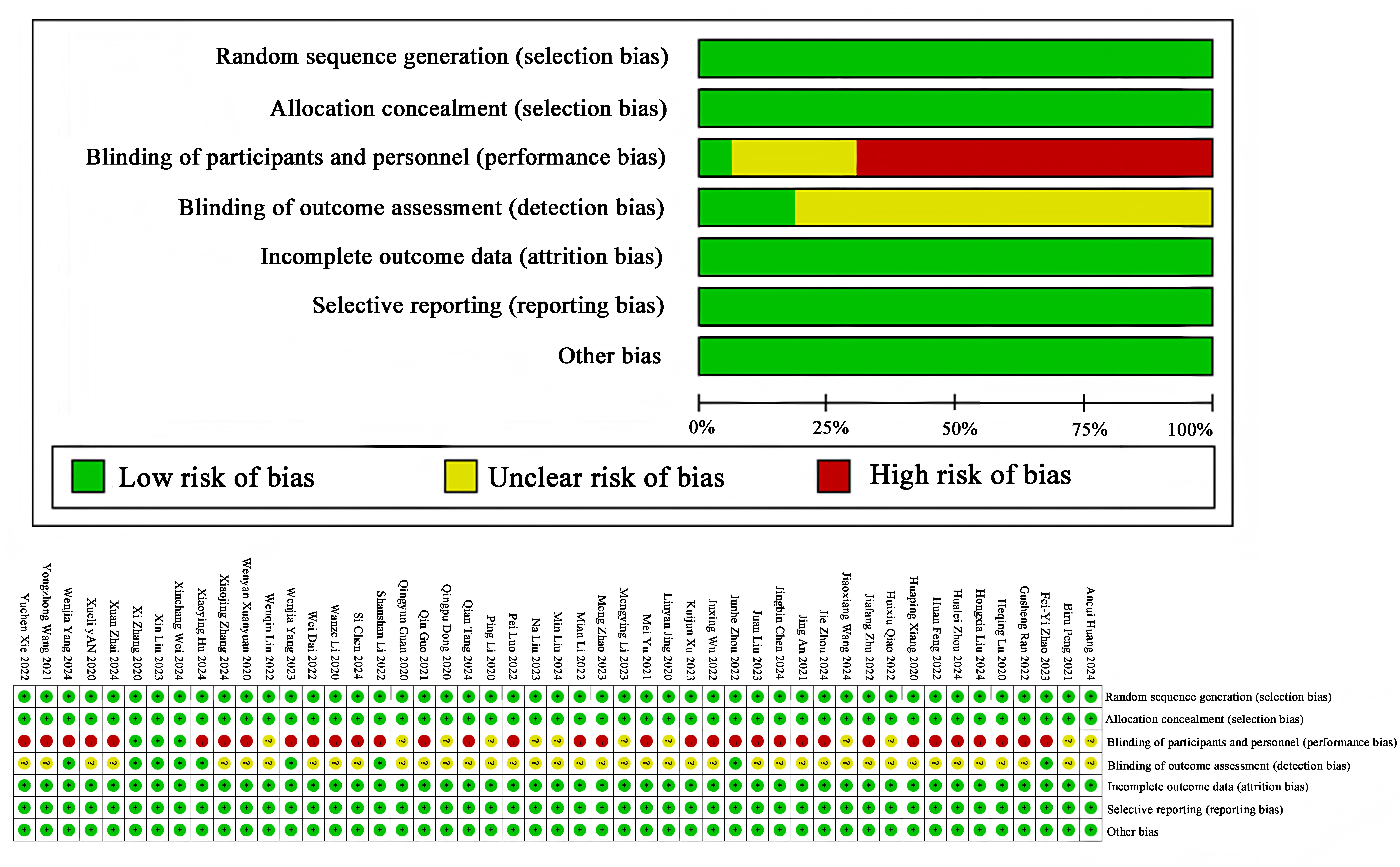

Supplement: Supplementary file 2 [file Image_1.jpeg]

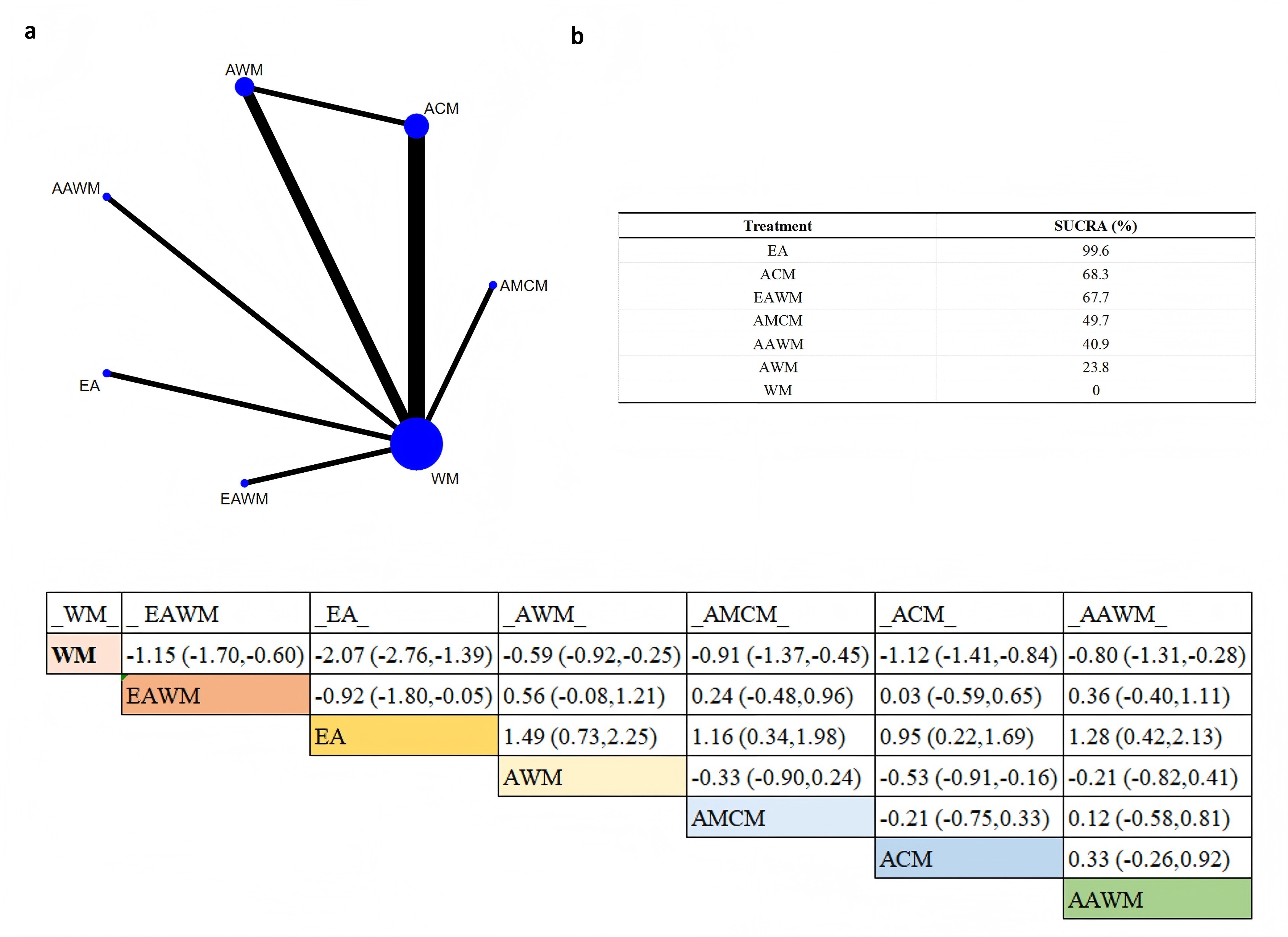

Supplement: Supplementary file 3 [file Image_2.jpeg]

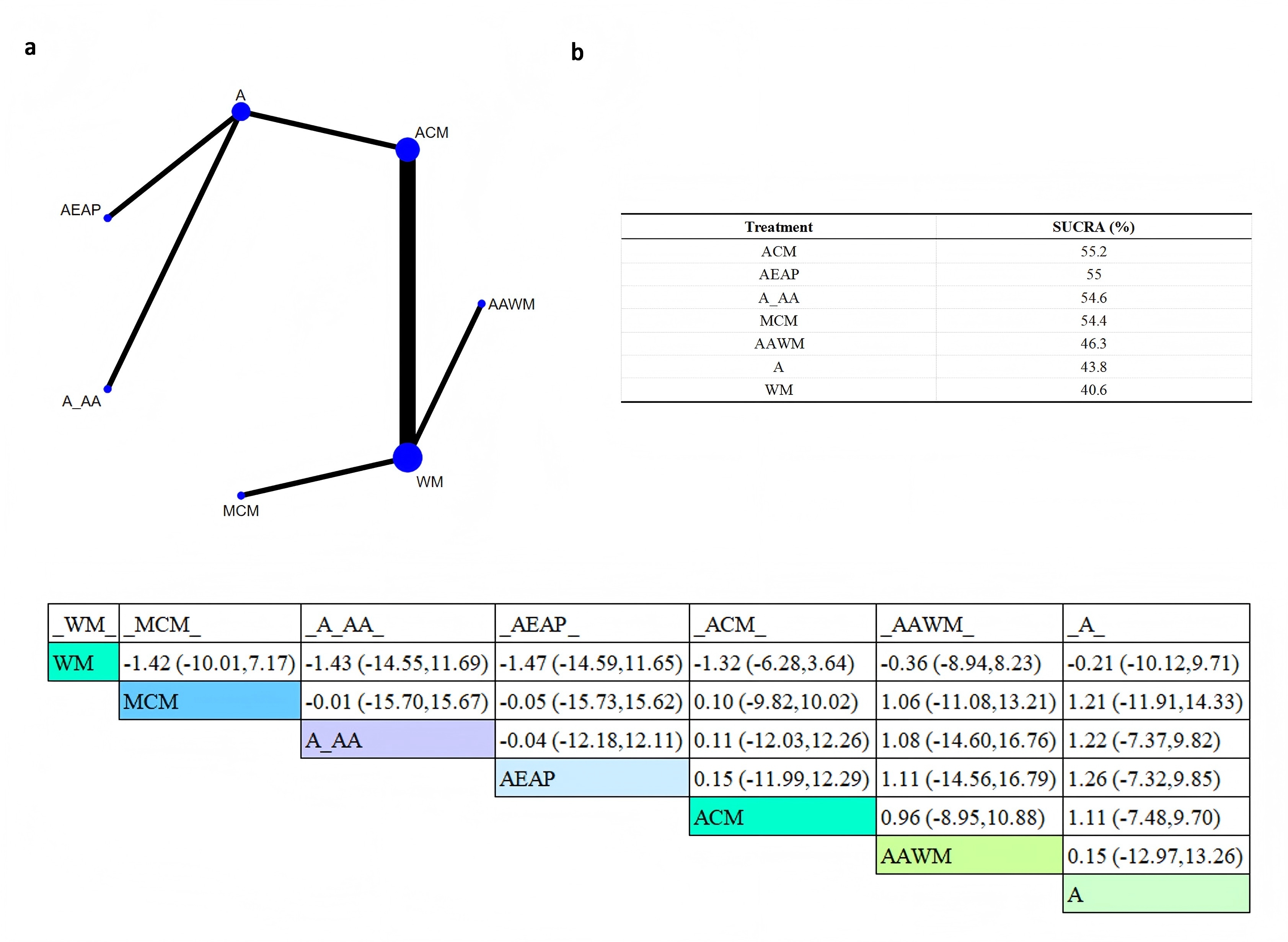

Supplement: Supplementary file 4 [file Image_3.jpeg]

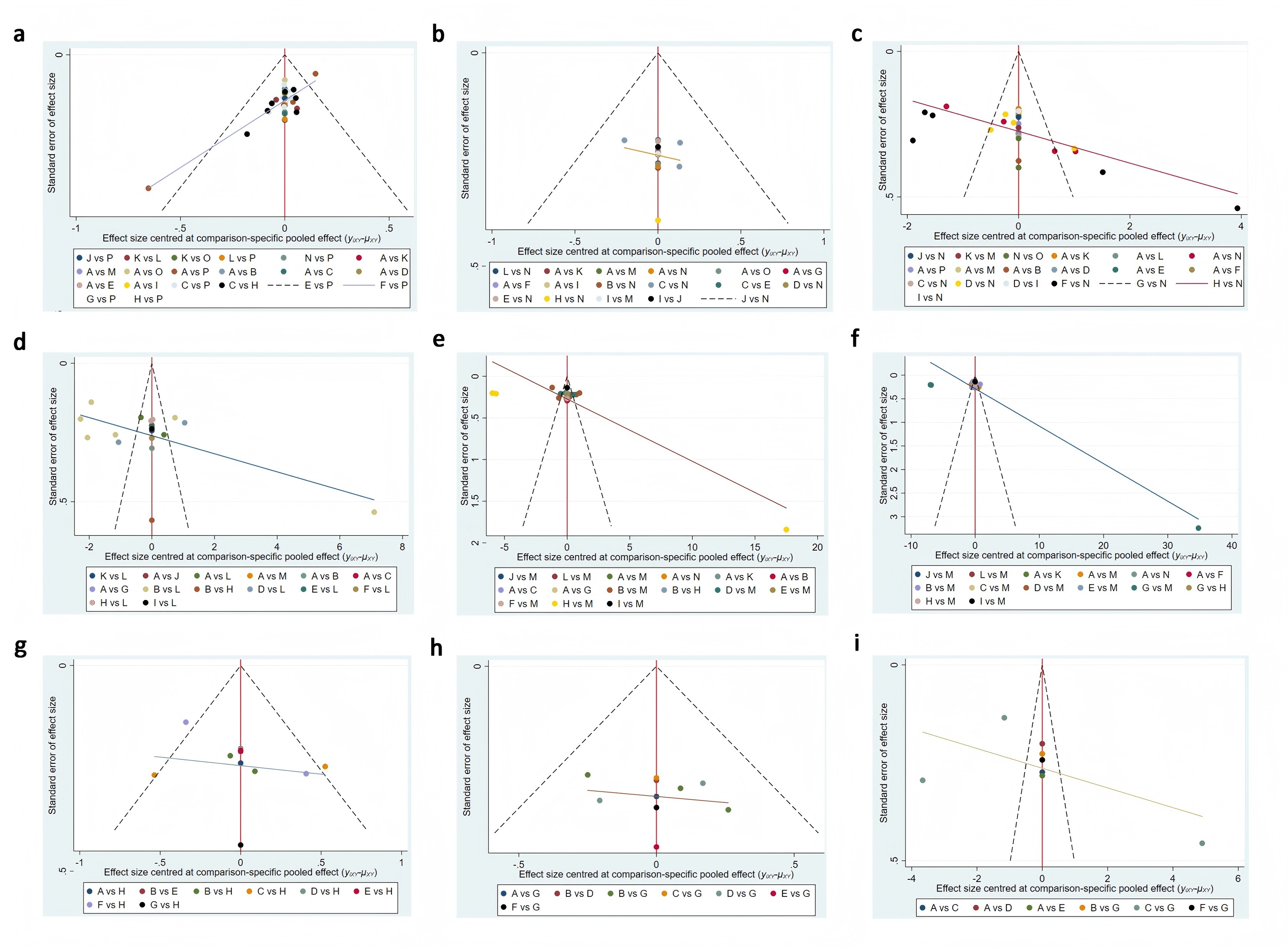

Supplement: Supplementary file 5 [file Image_4.jpeg]
